# Compliments

**Published:** 1879-01-20

**Authors:** 


### *COMPLIMENTS.*

We trust we shall be excused for an occasional allusion to what our friends say of us, or rather of our Journal. The compliments received are so many, that we have to deny ourselves the pleasure of special acknowledgment to the kind authors, and only mention one with our thanks, as a representative of the others.

A medical brother of Texas writes:—"Editors of THE RECORD: I have been a subscriber to six or seven Medical Journals, all of which are good and fully worth the subscription price; but I will say for you, without flattery, that THE RECORD is the best Journal for the busy practitioner I have ever seen. There is greater variety, less redundancy, and more practical information to the square inch than I have found in and other publication. I gladly renew my subscrption. I will continue to advocate the claims of your Journal. No practitioner should be without it."

Such expressions from our readers furnish gratifying evidence that our labor has not been in vain, and that the objects of THE RECORD, as proclaimed years ago, are being accomplished. These objects are: * * * First, to lay before the profession the truths of medical science, as they are being developed from month to month, in a condensed form for practical use. Second, to furnish an independent medium through which our medical brethren, especially in the South, may communicate gems of thought and experience now lost to medical literature. Third, to bring before our readers items of science, and all the new improvements in therapeutics, instruments, etc. Fourth, to call attention to standard works of authors, that they may read and be instructed thereby.

With these objects in view, we are satisfied our readers will extend to us and to the RECORD their aid and influence. We solicit original articles, reports of societies, clinics, news, etc., of interest to medical readers. Country physicians are especially invited to communicate their experience through the RECORD.

